# Oxidative Stress and Metabolic Perturbations in Wooden Breast Disorder in Chickens

**DOI:** 10.1371/journal.pone.0153750

**Published:** 2016-04-20

**Authors:** Behnam Abasht, Marie F. Mutryn, Ryan D. Michalek, William R. Lee

**Affiliations:** 1 Department of Animal and Food Sciences, University of Delaware, Newark, DE, United States of America; 2 Metabolon Inc., Durham, NC, United States of America; 3 Maple Leaf Farms, Leesburg, IN, United States of America; University of Insubria, ITALY

## Abstract

This study was conducted to characterize metabolic features of the breast muscle (pectoralis major) in chickens affected with the Wooden Breast myopathy. Live birds from two purebred chicken lines and one crossbred commercial broiler population were clinically examined by manual palpation of the breast muscle (pectoralis major) at 47–48 days of age. Metabolite abundance was determined by gas chromatography/mass spectrometry (GC/MS) and liquid chromatography coupled with tandem mass spectrometry (LC-MS/MS) using breast muscle tissue samples from 16 affected and 16 unaffected chickens. Muscle glycogen content was also quantified in breast muscle tissue samples from affected and unaffected chickens. In total, levels of 140 biochemicals were significantly different (FDR < 0.1 and fold-change A/U > 1.3 or < 0.77) between affected and unaffected chickens. Glycogen content measurements were considerably lower (1.7-fold) in samples taken from Wooden Breast affected birds when compared with samples from unaffected birds. Affected tissues exhibited biomarkers related to increased oxidative stress, elevated protein levels, muscle degradation, and altered glucose utilization. Affected muscle also showed elevated levels of hypoxanthine, xanthine, and urate molecules, the generation of which can contribute to altered redox homeostasis. In conclusion, our findings show that Wooden Breast affected tissues possess a unique metabolic signature. This unique profile may identify candidate biomarkers for diagnostic utilization and provide mechanistic insight into altered biochemical processes contributing to tissue hardening associated with the Wooden Breast myopathy in commercial chickens.

## Introduction

A novel muscle disease called Wooden Breast disease has emerged within the poultry industry significantly impacting the muscle health and meat quality of commercial broiler chickens. This disease, first described in the literature in 2014 by Sihvo et al. [1] is characterized by the severe hardening of the pectoralis major muscle during growth to market weight. The detection of severe cases of Wooden Breast disease can be performed and determined simply through manual palpation of the breast muscle. Although the underlying etiology of this disease remains to be determined, recent publications have shed light on the incidence rate [2], meat quality effects [3–5], and possible biological mechanisms and pathways [6] that contribute to the onset and course of the progression of this disease.

The incidence rate of Wooden Breast disease in commercial chickens is not yet well documented. However, Wooden Breast disease has been identified as an emerging quality defect with affected flocks reported to have up to 50% of individuals impacted [7]. This myopathy has been found in several countries around the world including the United States, Finland, Italy, and Brazil. The economic burden placed on the poultry industry because of Wooden Breast is likely to be great as the consequences of muscle abnormalities such as Wooden Breast and similarly White Striping, is a significant reduction in the aesthetic quality of broiler chicken meat [4].

Recent RNA-sequencing data performed on both Wooden Breast impacted birds and controls, established a characteristic gene expression profile for impacted birds. From their results, Mutryn et al. (2015) [6] established the likelihood of various contributing factors to the disease such as localized muscular hypoxia, oxidative stress damages to the affected muscle, and increased levels of intracellular calcium. It has also been suggested that excess production of decorin is another factor adding to the hardness of the pectoralis major potentially through an increase in collagen crosslinking [8]. It should also be noted that the authors’ hypothesized a deficiency in glucose metabolism due to the altered expression of multiple genes (e.g., asparagine synthetase and 6-phosphofructo-2-kinase/fructose-2,6-biphosphatase 3) [6]. Many authors also hypothesized high growth rate to be a contributing factor of this disease [4,6,8]. It has also been shown that high feed efficiency broiler chickens, which are both fast growing and have high breast muscle yield, are more likely to develop Wooden Breast disease [2]. Comparisons between two commercial broiler lines showed that there are higher incidences of myopathies in chickens from a line selected for high breast muscle yield compared to chickens from a line selected for moderate breast muscle yield [9].

In this study, we aim to characterize the biochemical signature of Wooden Breast disease through high throughput metabolomics profiling of breast muscle isolated from affected and unaffected chickens. The results define biomarkers of myopathy in affected birds and reveal major metabolic differences between affected and unaffected chickens. In addition to confirming the previously hypothesized glucose metabolism deficiency, the results of this study shed light on novel contributing factors to this disease. To the best of our knowledge, this is the first study conducted using metabolomics profiling of a muscle disorder, specifically Wooden Breast, in chickens.

## Materials and Methods

### Birds

Chickens obtained from Heritage Breeders (Princess Anne, MD) were sampled across two genetically distinct purebred lines (lines 1 and 2) and one commercial broiler (CB) population. The CB population is the result of crossing three purebred lines, two of which (lines 1 and 2) are included in this study. To identify Wooden Breast-affected and unaffected chickens, live birds were clinically examined by manual palpation of the breast muscle (pectoralis major) at 47–48 days of age. Birds with severe or mildly severe hardness of the breast muscle were identified as affected, whereas chickens with no physical signs of hardness in the breast muscle were considered to be unaffected. The affected chickens sampled for this study consisted of birds whose whole breast muscle was affected. This was also confirmed at necropsy by visual observation of macroscopic lesions, specifically areas of firm, swollen, and discolored muscle tissue on pectoralis major muscle. Although white-striping occurrence was not systematically assessed in the current study, it should be noted that all Wooden Breast affected chickens appeared to have some degree of white striping. After euthanasia by cervical dislocation, breast muscle tissue was harvested from 16 unaffected and 16 affected birds (4 unaffected and 4 affected samples from line 1 and BC population; and 8 unaffected and 8 affected samples from line 2), immediately flash frozen in liquid nitrogen, and stored at -80°C until further processing. The University of Delaware Agricultural Animal Care and Use Committee approved the animal protocol used for this scientific study.

### Metabolomics Analysis

Tissue samples from the breast muscle of 16 affected and 16 unaffected chickens from lines 1 and 2 and CB population were ground by hammering at frozen state using liquid nitrogen, and frozen aliquots of samples were shipped on dry-ice overnight to Metabolon Inc. (Durham, NC) for metabolomics profiling. The platform and methodology used by Metabolon Inc. for metabolomics profiling has been previously described by Evans et al., (2009)[10] and Reitman et al. (2011)[11]. This platform utilizes a large, well-annotated spectral library generated from more than 4000 authentic standards [10,11]. Numeric representations of the spectral entries for the metabolites identified in our sample set are provided in S1 File.

### Glycogen Extraction of Muscle Tissue

Glycogen extraction was performed and slightly modified based on instructions provided by Abcam®, prior to use of the Abcam® Glycogen Assay Kit. Both glycogen extraction and the glycogen assay were performed on 24 breast muscle samples from lines 1 and 2 and CB population including 12 samples affected with Wooden Breast and 12 unaffected samples. All but two samples used in the glycogen assays were the same samples used in the metabolomics experiment. We ran out of tissue samples for two unaffected birds from CB population, and therefore, tissue samples from 2 other unaffected CB birds were used in the glycogen assay. The line 2 samples were random samples of 8 affected and 8 unaffected samples used in the metabolomics experiment.

Glycogen extraction was performed on roughly 80mg of frozen muscle tissue which was placed into a test tube containing 300ul of 30% KOH. These tubes were heated at 100°C for 2 hours. After cooling, 900 ul of 95% ethanol was added to each tube, and then all tubes were centrifuged for 10 minutes at 3,000 rpm to precipitate the crude glycogen. The supernatant was discarded, and the precipitate was dissolved in a minimum amount of dH_2_0 and then acidified to a pH of 3 with 5N HCl. Three volumes of ethanol was used for reprecipitation for washing steps which were repeated two times. The supernatant was discarded, and the precipitate was dried under a ventilation hood until all supernatant was evaporated. The dried material was resuspended and dissolved in 300ul of dH_2_0. Before proceeding to the glycogen assay, each glycogen extraction sample was diluted 20x.

### Glycogen Assay

The glycogen assay was performed based on the manufacturer’s instructions using Abcam® Glycogen Assay Kit. For this assay, the colorimetric parameters were used, with a clear 96 well plate. The sample volume for each well was 20ul. Measurements were set at OD = 570nm using the SpectraMax® M2 (Molecular Devices). Data analysis was also performed based on instructions by the manufacturer, using the standard curve to calculate the final concentration of glycogen in mg/g.

### Statistical Analysis

Following median scaling, log transformation and imputation of missing values with the minimum observed value for each compound, data were analyze using “Response Screening Platform” in JMP Pro 11. The “Response Screening Platform” performs tests across a large number of responses. We used the “robust estimate” method of this platform, in order to minimize the sensitivity of statistical tests in regards to outliers. To maximize statistical power, data was analyzed across purebred lines 1 and 2 and CB population, i.e., 16 Wooden Brest-affected *vs*. 16 unaffected birds, rather than within each line or population. Biochemical compounds with an FDR adjusted p-value of < 0.1 and a fold-change greater than 1.3 were considered significant. Biological pathways were assigned to each metabolite, allowing the examination of overrepresented pathways. Random forest analysis (RFA) [12,13] was conducted using an R package [14] to determine which variables (biochemicals) made the largest contribution to the classification, with “Mean Decrease Accuracy” (MDA) as a measure of “variable importance”. For each variable, we determined the MDA by randomly permuting the variable, running the observed values through the “trees” and then reassessing the prediction accuracy. The mean values of log-transformed median-scaled metabolite concentration in affected and unaffected birds across all three populations were used to calculate fold-change differences between affected and unaffected birds. Student's t-test was used for statistical comparisons of the difference in mean glycogen content between Wooden Breast-affected and unaffected muscle samples.

## Results

The complete dataset received from Metabolon Inc. comprised a total of 282 compounds of known identity. All original data and log-transformed median-scaled values are provided in S2 File. Data analysis using the “Response Screening Platform” in JMP identified 140 compounds with an FDR adjusted p-value of < 0.1 and a fold-change greater than 1.3 between affected and unaffected samples.

**Fig 1:**
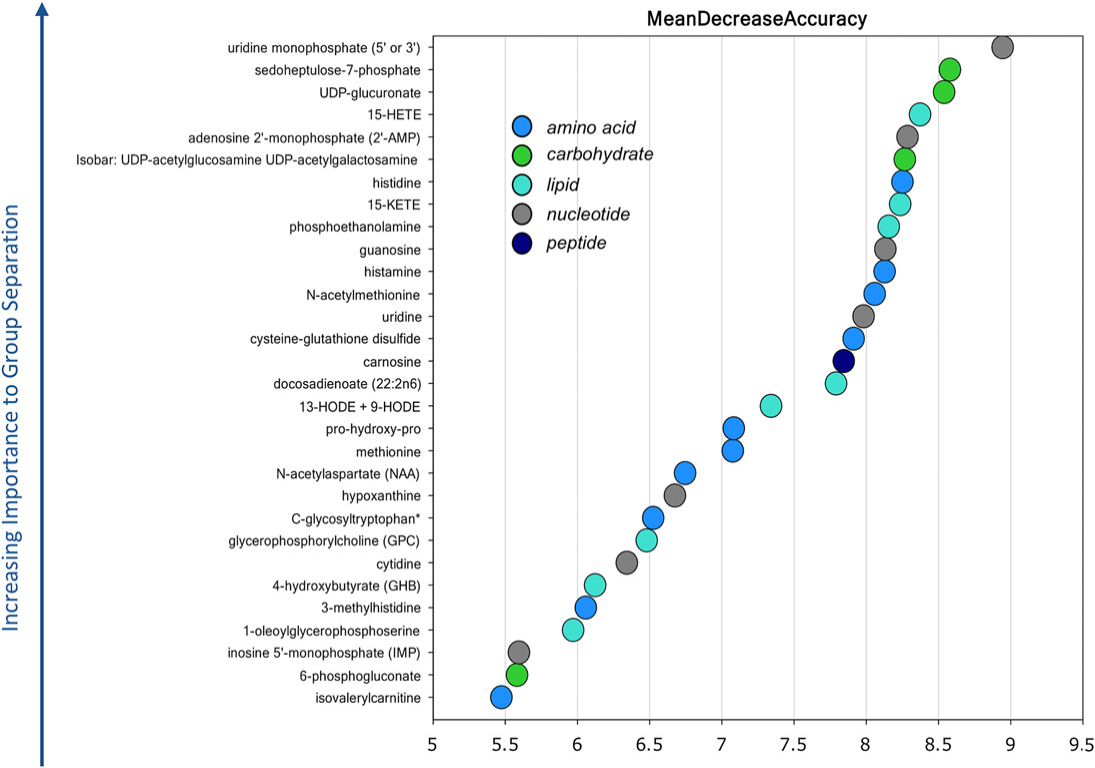
Top 30 Metabolites Based on their Importance for Separating the Groups (Affected and Unaffected Tissues). Random forest analysis (RFA) was utilized to determine which variables (biochemicals) had the largest contribution on classification. “Mean Decrease Accuracy” (MDA) was used as a measure of “variable importance”.

**Fig 2:**
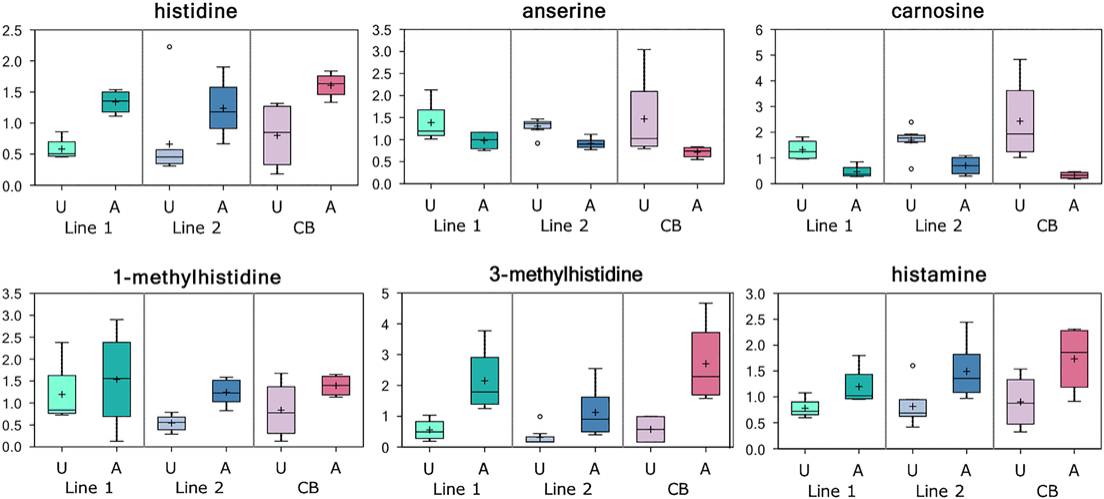
Histidine Metabolism in Affected Tissues. Levels (median-scaled and log-transformed) of histidine and other related metabolites in breast muscle tissue samples obtained from affected (A) and unaffected (U) chickens that were sampled from two genetically distinct purebred lines (lines 1 and 2) and a commercial broiler (CB) population. Elevated histidine levels in affected tissue were accompanied by an accumulation of histamine, 1-methylhistidine, and 3-methylhistidine, which may reflect skeletal muscle degeneration and oxidative stress. In contrast, the histidine derived antioxidants carnosine and anserine, were depleted in affected breast muscle tissue suggesting altered redox homeostasis.

Random forest analysis based on the biochemicals detected in this study identified a distinct biochemical signature between affected and unaffected tissues. All affected samples were also predicted as being affected by RFA (i.e., predictive accuracy of 100%; Table 1) suggesting that these metabolites are of interest as biomarkers for identifying this condition. However, two unaffected samples, Sample 51 from line 2 and Sample 47350 from CB, were predicted as being affected by RFA (Table 1). These two samples were also closely clustered with affected samples based on their gene expression profile, which was previously obtained using RNA-sequencing [6] (Sample 51) and Nanostring technology [15] (Samples 51 and 47350). As explained before, these samples may represent instances of low-level (asymptomatic) myopathy [6,15]. Fig 1 provides the top 30 metabolites ranked based on their importance for distinguishing between affected and unaffected groups. Comparisons between metabolite levels and glycogen content between affected and unaffected samples will be discussed in the following paragraphs, as well as their potential biological roles in the Wooden Breast myopathy (see Discussion section). Compared with the unaffected controls, several amino acids were significantly elevated in the affected muscle samples (Fig 2). Specifically, elevated histidine levels (fold-change = 2.26) in the affected tissue were accompanied by an accumulation of the related metabolites histamine (fold-change = 2.02), 1-methylhistidine (fold-change = 2.01), and 3-methylhistidine (fold-change = 4.68). The levels of branched chain amino acids valine (fold-change = 1.50), isoleucine (fold-change = 1.37), and leucine (fold-change = 1.51); the proline-related metabolite pro-hydroxy-pro (fold-change = 2.51) were also higher in affected tissues.

**Table 1:** Random Forest Analysis (RFA), Wooden Breast Affected *vs*. Unaffected Chickens.

	*Predicted group*	
*Actual group*	Unaffected	Affected
Unaffected	14	2
Affected	0	16

**Fig 3:**
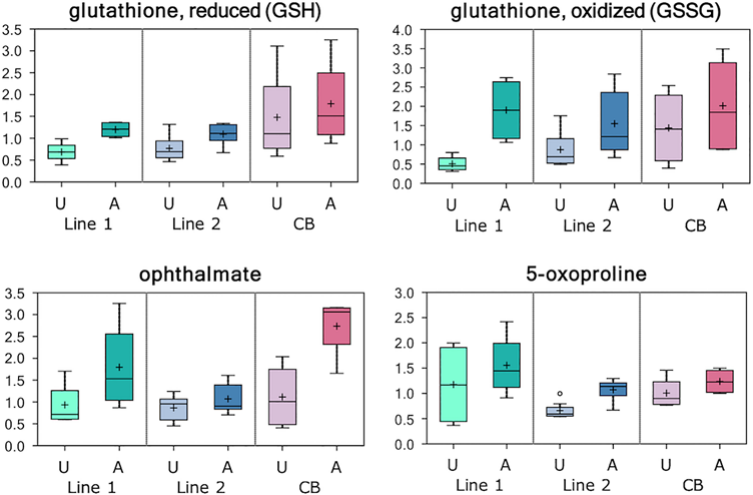
Glutathione Metabolism in Affected Tissues. Levels (median-scaled and log-transformed) of glutathione and other related metabolites in breast muscle tissue samples obtained from affected (A) and unaffected (U) chickens that were sampled from two genetically distinct purebred lines (lines 1 and 2) and a commercial broiler (CB) population. Higher levels of the cysteine derived metabolites glutathione (GSH) and oxidized glutathione (GSSG) may reflect free radical exposure. These tissues also possessed higher levels of cysteine-glutathione disulfide (historically a marker of oxidative stress), the gamma-glutamyl amino acid catabolite 5-oxoproline that may suggest import of extracellular glutathione, and tripeptide ophthalmate that is an analogue of glutathione often produced in response to increased ROS and glutathione depletion.

The histidine-derived antioxidants carnosine (fold-change = 0.37) and anserine (fold-change = 0.75) were decreased, and glutathione metabolites, glutathione (GSH; fold-change = 1.67) and glutathione disulfide (GSSG; fold-change = 2.17) were increased in affected tissues (Fig 3). Furthermore, affected tissues exhibited elevated levels of the tripeptide ophthalmate (fold-change = 2.03).

Affected tissues also possessed higher levels of cysteine-glutathione disulfide (fold-change = 2.89) and the gamma-glutamyl amino acid catabolite 5-oxoproline (fold-change = 1.57). Cysteine-glutathione disulfide and carnosine are among the top important metabolites for separating affected and unaffected tissues by RFA (Fig 1).

The affected muscle tissue exhibited reduced levels of the glycolytic intermediates glucose 6-phosphate (G6P; fold-change = 0.46) and fructose 6-phosphate (fold-change = 0.43) as well as the end-products lactate (fold-change = 0.68) and pyruvate (fold-change = 0.43). Consistent with reduced glycolytic metabolites, the affected muscle showed a significant (*p* < 0.001) reduction in muscle glycogen content (fold-change = 0.59; S3 File).

The pentose phosphate pathway (PPP) was also altered in Wooden Breast affected birds, as demonstrated by the accumulation of 6-phosphogluconate (fold-change = 2.84) and sedoheptulose 7-phosphate (fold-change = 3.73). The latter is the second most important metabolite for distinguishing affected and unaffected tissues by RFA (Fig 1). Finally, elevated levels of fumarate (fold-change = 1.93) and malate (fold-change = 1.87) in affected breast muscle marked an imbalance in the TCA cycle.

Ascorbate (fold-change = 1.85) and nucleotide sugars UDP-glucuronate (fold-change = 4.89), isobar UDP-acetylglucosamine and UDP-acetylgalactosamine (fold-change = 2.88) were among the top 10 metabolites for differentiating affected and unaffected tissues (Fig 1).

**Fig 4:**
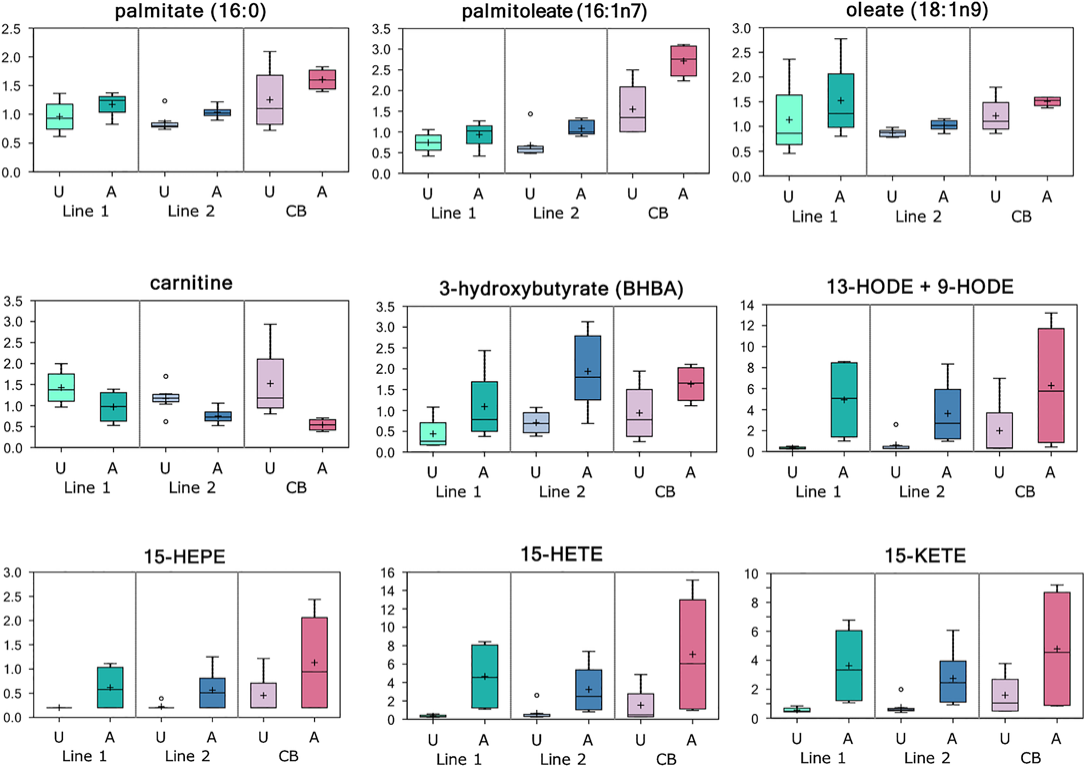
Deregulated Lipid Metabolism in Affected Tissues. Levels (median-scaled and log-transformed) of lipid and lipid metabolism related metabolites in breast muscle tissue samples obtained from affected (A) and unaffected (U) chickens that were sampled from two genetically distinct purebred lines (lines 1 and 2) and a commercial broiler (CB) population. Multiple long chain fatty acids including palmitate, palmitoleate, stearate, and oleate accumulated in affected muscle. Increased lipid availability may support mitochondrial β-oxidation as lower levels of free carnitine may reflect utilization for long chain fatty acid transport into the mitochondria. Furthermore, elevated levels of the ketone body 3-hydroxybutyrate (BHBA) have historically been a marker of lipid oxidation. Increased lipid availability may also consequently facilitate the generation of lipid peroxidation products such as 13-HODE and 9-HODE. These metabolites are often indicative of free radical exposure. The eicosanoids 15-HEPE, 15-HETE, and 15-KETE were also elevated in affected tissues and may represent a compensatory mechanism to balance inflammation since these lipid mediators have been shown to exhibit anti-inflammatory properties.

Multiple long chain fatty acids including palmitate, palmitoleate, stearate, and oleate accumulated in the affected muscle (Fig 4). The levels of the phospholipid catabolites glycerol 3-phosphate (fold-change = 2.24), glycerophosphoethanolamine (fold-change = 2.03), and glycerophosphorylcholine (fold-change = 2.17) were also higher in affected tissues as compared with controls. Furthermore, affected tissue showed higher levels of glycerol (fold-change = 1.42) and monoacylglycerols, including 2-linoleoylglycerol (fold-change = 1.33) and 1-palmitoylglycerol (fold-change = 1.57), and elevated levels of the ketone body 3-hydroxybutyrate (BHBA; fold-change = 2.69).

Lipid peroxidation products such as 13-HODE and 9-HODE (13-HODE + 9-HODE; fold-change = 5.25) as well as the eicosanoids 15-HEPE (fold-change = 2.30), 15-HETE (fold-change = 6.05), and 15-KETE (fold-change = 4.21) were also elevated in affected tissues. Of these three eicosanoids, 15-HETE and 15-KETE are among top important metabolites for distinguishing affected and unaffected muscles (Fig 1).

Finally, the levels of nucleotides and their related metabolites were significantly different between affected and unaffected birds. Compared with controls, affected tissues had higher levels of cytidine (fold-change = 1.89), thymidine (fold-change = 1.56), adenine (fold-change = 1.52), uridine (fold-change = 1.72), and guanosine (fold-change = 1.57) and lower levels of their related metabolites inosine 5' monophosphate (IMP; fold-change = 0.63), adenosine 5'-diphosphate (ADP; fold-change = 0.58), adenosine 5'-monophosphate (AMP; fold-change = 0.53) and cytidine-3'-monophosphate (3'-CMP; fold-change = 0.68). Affected tissue also showed higher levels of the compounds of nucleotide catabolism such as hypoxanthine (fold-change = 3.43), xanthine (fold-change = 1.72), urate (fold-change = 3.22) and uracil (fold-change = 2.97). Notably, of top 30 important metabolites for separating affected and unaffected tissues by RFA, seven are involved in nucleotide metabolism (Fig 1).

## Discussion

### Amino Acid Metabolism and Oxidative Stress

Elevated histidine levels in the affected tissue were accompanied by an accumulation of the related metabolites histamine, 1-methylhistidine, and 3-methylhistidine. Previous studies demonstrated that increased histamine levels are often associated with pain and inflammation [16–18]. Furthermore, these findings may reflect muscle degeneration considering that 1-methylhistidine can be indicative of skeletal muscle oxidative stress, and 3-methylhistidine is an established biomarker of muscle protein breakdown [19,20]. Similarly, high levels of the branched chain amino acids valine, isoleucine, and leucine, and of the proline-related metabolite pro-hydroxy-pro in the affected muscle can be indicators of extracellular matrix remodeling associated with change in muscle tissue [21,22]. Changes in the muscle tissue of Wooden Breast chickens can also be supported by the presence of fibrosis containing collagen-rich tissues [1,3,5,8].

Decreased levels of the histidine-derived antioxidants carnosine and anserine in the affected tissues suggest an altered redox homeostasis. In agreement, high levels of glutathione metabolites, glutathione and glutathione disulfide, may be indicative of an increase in total glutathione availability that may arise from a change in synthesis and/or import, and can support free radical detoxification. These results are also consistent with elevated levels of taurine and the tripeptide ophthalmate in affected tissues. Ophthalmate is an analog of glutathione arising from cysteine depletion and is often elevated in response to increased reactive oxygen species (ROS) exposure [23].Taurine is a sulfur-containing amino acid usually produced in tissues exposed to high levels of oxidants [24].

Higher levels of cysteine-glutathione disulfide in affected tissues may reflect free radical exposure, and higher levels of the gamma-glutamyl amino acid catabolite 5-oxoproline in affected tissues suggests the import of extracellular glutathione. Collectively, these findings strongly suggest that affected chickens possess biological markers of muscle degradation and oxidative stress.

### Carbohydrate Metabolism

Reduced levels of the glycolytic intermediates glucose 6-phosphate (G6P) and fructose 6-phosphate and of the end-products lactate and pyruvate in affected samples are consistent with a reduction in muscle glycogen content. These results can also be supported by a previous study conducted on chickens impacted with white striping, a meat quality disorder impacting integrity due to fatty striations in breast muscle tissue, which also found reduced levels of glycolytic enzymes [3]. These changes in glycogen content and glycolysis may arise from a change in glucose utilization rather than glucose availability. There is evidence to suggest this as sorbitol, which is elevated in the affected muscle (fold-change = 1.88), is often generated through the reduction of excess glucose (see below the section on sorbitol accumulation) [25].

In vitro studies showed that G6P is an allosteric activator of glycogen synthase (GS) [26,27], which may partly explain why breast muscle samples from affected chickens have low glycogen content. A significantly higher ultimate pH (pH at 24 hours postmortem) in affected tissues may also be related to glycogen depletion and reduced glycolytic potential within affected muscle [2]. These observation has also been previously confirmed by Mudalal et al. (2014) [4], as Wooden Breast and White Striping-affected chickens exhibited higher ultimate pH values of the breast muscle when compared to unaffected birds. They suggested that these abnormalities might potentially reduce the levels of glycogen in the muscle [4]. As previously reported by Berri et al. (2001 & 2011), postmortem lactate production, which is a major factor influencing ultimate pH, can be limited due to low glycogen content of the muscle [28,29].

White Striping in broiler chickens shows some overlapping histological characteristics with Wooden Breast disease [1]. Although white-striping occurrence was not systematically assessed in the current study, all Wooden Breast-affected chickens appeared to have some degree of white striping. Collectively, these findings may indicate that in addition to histological and microscopic features, White Striping and Wooden Breast disease may share some common metabolic characteristics.

An increase in PPP metabolites 6-phosphogluconate and sedoheptulose 7-phosphate may be consistent with reduced glycolysis as this pathway may utilize glucose. The PPP facilitates NADPH regeneration for anabolic growth and glutathione reduction. Evidence in the literature demonstrates that muscle fibers from patients with congenital myopathies, inflammatory myopathies, metabolic myopathies, and endocrine myopathies display an enhanced activity of PPP enzymes [30]. Thus, these findings suggest an elevated PPP flux in affected tissues in order to facilitate muscle growth and regeneration, as well as free radical detoxification. Finally, elevated levels of fumarate and malate in affected breast muscle marked an imbalance in the TCA cycle. These observations may warrant further investigation, considering that fumarase deficiency can be characteristic of multiple myopathies [31].

### Sorbitol Accumulation

Accumulation of sorbitol in Wooden Breast-affected tissues may provide important insights into the pathogenesis of this muscle disorder. Sorbitol is an organic osmolyte, and its accumulation can increase intracellular osmolality leading to aberrant cellular osmoregulation [32]. Damaging effects of intracellular sorbitol build-up have been observed in the eye lens, where it can cause cell swelling, ultimately leading to changes in cell membrane permeability and biochemical changes associated with the formation of a cataract [33]. Also, it has been previously found that in the rabbit lens and human erythrocytes, the sorbitol biosynthesis pathway accounts for 33% and 11% respectively, of total glucose used during hyperglycemia [25]. In this pathway, glucose is reduced to sorbitol, and NADPH serves as a source of electrons for this reduction. Since NADPH is also required for the regeneration of reduced glutathione (GSH), increases in sorbitol biosynthesis has the potential to exacerbate intracellular oxidative stress in affected muscle [34].

### Nucleotide Sugar Metabolism

Nucleotide sugars are utilized in glycosylation reactions to generate glycosaminoglycans such as chondroitin, dermatan, heparin and hyaluronan [35,36]. The majority of glycosaminoglycans are covalently attached to core proteins, forming glycosylated protein complexes known as proteoglycans. Since levels of nucleotide sugars are increased in the affected muscle, it is likely that these metabolites are elevated to support or promote pathological excessive ECM remodeling through the formation of glycosaminoglycans and proteoglycans.

A recent study by Velleman and Clark (2015) showed increased *decorin* expression in Wooden Breast samples from a purebred line of commercial broiler chickens [8]. The protein encoded by this gene is a proteoglycan composed of a protein core containing leucine repeats attached to a glycosaminoglycan chain, either chondroitin sulfate or dermatan sulfate. In support of this previous study, our study on gene expression analysis using RNA-sequencing shows higher expression of *decorin* in affected muscle compared with controls in all 3 populations studied here (unpublished). Velleman and Clark (2015) [8] hypothesized that higher levels of decorin production would lead to increased collagen crosslinking in Wooden Breast disease.

Moreover, *hyaluronan synthase 2* (*HAS2)*, a gene encoding a highly active isoform of hyaluronan synthase [37], is expressed at greater levels in the affected tissues compared with controls in all 3 populations (line 1 and 2 and CB population; fold change = 2.2 to 3.3; unpublished). Hyaluronan synthases use UDP-acetylglucosamine and UDP-glucuronate as substrates to produce hyaluronan. Since the levels of substrates and the expression of *HAS2* are elevated in the affected muscle, we expect higher levels of hyaluronan to be produced in the breast muscle of Wooden Breast-affected birds. In agreement, a previous study has shown that hyaluronan production is increased in an *in vitro* cellular model expressing higher levels *HAS2* and *UDP-glucose dehydrogenase* (*UGDH*) [38]. To elucidate the connection between hyaluronan accumulation and increased *UGDH expression*, it should be noted that the enzyme encoded by this gene converts UDP-glucose to UDP-glucuronate, a substrate for hyaluronan production by hyaluronan synthases (HAS1, HAS2 and HAS3).

Hyaluronan is one of the main components of the extracellular matrix. In addition to its function as a structural component, hyaluronan plays important roles in cell proliferation, migration and inflammation [39]. A study by Li et al., (2015) has shown that the overexpression of *HAS2* increases hyaluronan production and cell proliferation and is correlated with the tumorigenesis and metastasis of human breast cancer [40]. Another study has suggested a link between induced hyaluronan accumulation and increased hyaluronan crosslinking as well as changes in vascular smooth muscle cells phenotype and proliferation [41]. It has also been found that hyaluronan plays a role in inflammation and is involved in leukocyte recruitment, inflammatory cell activation, as well as cytokine release [42]. Increased expression of hyaluronan synthases was reported to promote monocyte adhesion [43]. In light of these previous results, it can be hypothesized that the expected increase in hyaluronan production would play an important role in Wooden Breast pathology. Further studies are needed to measure hyaluronan content of the breast muscle from affected and unaffected chickens and investigate potential roles of this glycosaminoglycan in rapid muscle growth, inflammation and infiltration of inflammatory cells in Wooden Breast disease.

**Fig 5:**
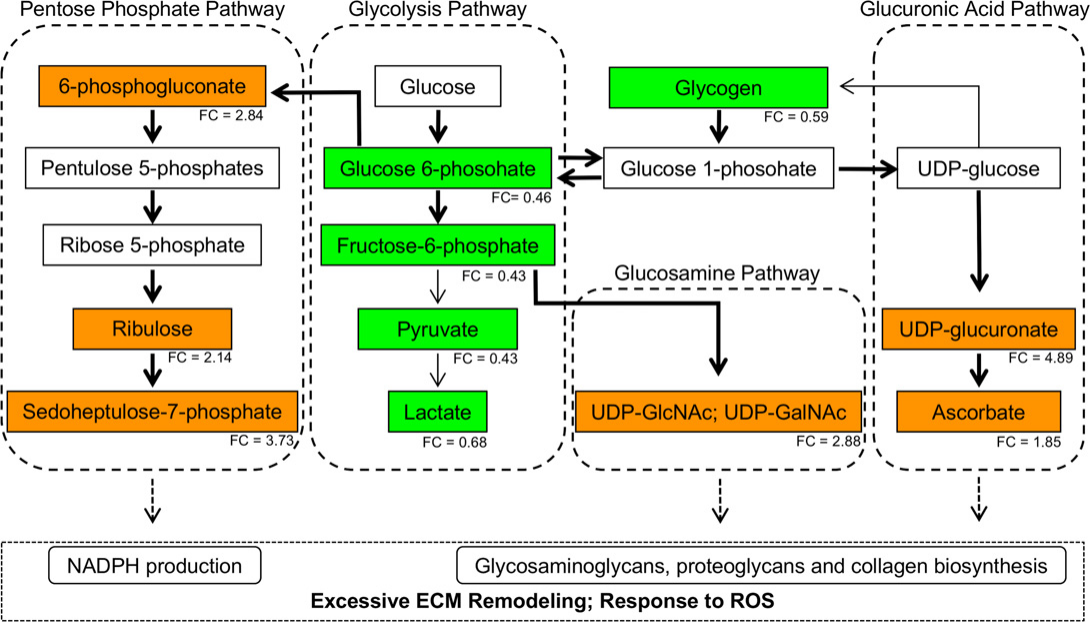
Altered Carbohydrate Metabolism in Wooden Breast Disease. The affected tissues exhibited evidence of altered glycogen metabolism and glucose utilization that resulted in the accumulation of multiple intermediates of the pentose phosphate, glucosamine and glucuronic acid pathways. These changes collectively indicate a rerouting of carbohydrate flux from glycolysis to these metabolic pathways in order to combat oxidative stressors and support detoxification, regeneration and excessive remodeling in the affected tissues. Metabolites (or glycogen) presented in the orange and green boxes were at significantly higher and lower levels in Wooden Breast, respectively. The numeric values under the colored boxes represent the fold-change (FC) differences in metabolite levels or glycogen content between affected (A) and unaffected (U) tissues (A / U). The tick arrows represent the potentially most active biochemical reactions and pathways.

Elevated levels of nucleotide surges (UDP-glucuronate and isobar UDP-acetylglucosamine and UDP-acetylgalactosamine) in the affected muscle suggest an increase in the activity of pathways generating these metabolites, mainly hexosamine and glucuronic acid pathways. Increased activity of hexosamine and glucuronic acid pathways would enhance glucose shunting into these pathways, reducing glucose availability for glycogen synthesis and glycolysis. Fig 5 presents a model highlighting the connection between carbohydrate and nucleotide sugar metabolism and their possible association with oxidative stress and excessive ECM remodeling in Wooden Breast disease.

Elevated levels of UDP-glucuronate may also be closely related to the high levels of ascorbate in the affected tissues, as UDP-glucuronate is an intermediate in ascorbate biosynthesis. Ascorbic acid (Vitamin C) is one of the most crucial antioxidants in mammalian and avian tissues. It serves as an electron donor in a variety of enzymatic reactions and is essential for the biosynthesis of collagen [44]. The role played by ascorbic acid in the biosynthesis of collagen can be of critical importance because fibrosis, which is a prominent feature of Wooden Breast [1], acts to replace wasted muscle tissue with collagen and proteoglycans. Increased collagen synthesis in Wooden Breast has also been previously supported by RNA-sequencing results showing higher expression of several genes within the collagen family in the affected muscle compared with controls [6].

Results from previous studies have shown that ascorbate biosynthesis in animals is tightly coupled to carbohydrate metabolism, with glycogen being the main source of glucose 1-phosoate needed for *de novo* ascorbate synthesis [45]. In mice and rats, carbohydrate restriction is associated with a decrease in the hepatic ascorbate content [46]. Furthermore, Braun et al., (1994) showed that ascorbate biosynthesis is absent in hepatocytes depleted of glycogen reserves [47]. Given the results of these previous studies it is reasonable to hypothesize that the glycogen depletion in Wooden Breast-affected muscle may be secondary or partially due to an over-activation of the ascorbate biosynthesis pathway. A further consequence of increased glucose flux through the ascorbate biosynthesis pathway is the production of excess H_2_O_2_, a byproduct of ascorbate biosynthesis. This can potentially consume glutathione and exacerbate oxidative stress in the affected tissues [48]. Therefore, it may be hypothesized that vitamin C supplementation of the commercial chicken diet could reduce the activity of this pathway and its potential damages to muscle. It is interesting to note that ascorbate-synthesizing ability has been lost in primates and several other species. An evolutionary selective advantage of this loss has been thought to be related to glutathione consumption by this pathway [45].

### Lipid Metabolism

Elevated lipid levels in the affected muscle may suggest a change in membrane dynamics, as demonstrated by higher levels of the phospholipid catabolites glycerol 3-phosphate, glycerophosphoethanolamine, and glycerophosphorylcholine in affected tissues. Previously, increased levels of fatty acids were found in the breast muscle tissue of chickens affected by white striping, suggesting that this increase may be characteristic and common to both of these recently reported types of muscle quality degradation [49]. Furthermore, higher levels of glycerol in the affected muscle may reflect the hydrolysis of complex lipids such as triglycerides, as supported by the accumulation of multiple monoacylglycerols, including 2-linoleoylglycerol and 1-palmitoylglycerol. Increased lipid availability may support mitochondrial oxidation as lower levels of free carnitine may reflect utilization for long chain fatty acid transport into the mitochondria. Also, elevated levels of the ketone body 3-hydroxybutyrate (BHBA) are generated from excess acetyl CoA and can serve as a marker of lipid oxidation. Consequently, increased lipid availability may also facilitate the generation of lipid peroxidation products such as 13-HODE and 9-HODE. These metabolites are often indicative of free radical exposure and are PPAR (peroxisome proliferator-activated receptor) ligands [50] associated with atherosclerosis progression and inflammatory hyperalgesia that can typically accompany myopathy. The elevated levels of eicosanoids 15-HEPE, 15-HETE, and 15-KETE in affected tissues may represent a compensatory mechanism to balance inflammation as these lipid mediators have been shown to exhibit anti-inflammatory properties. It has also been shown that, 15-KETE plays a role in cell-cycle progression and cell migration and mediates excessive proliferation and migration of pulmonary artery endothelial cells [51].

### Nucleotide Metabolism

Elevated PPP metabolite levels in affected tissues may suggest a greater capacity for nucleic acid synthesis, as potentially demonstrated by the accumulation of cytidine, thymidine, adenine, uridine, and guanosine as compared with the controls. Alternatively, these differences may reflect decreased utilization due to enhanced ADP/AMP/CMP/IMP turnover. Indeed, lower levels of these metabolites and elevated levels of the related catabolites hypoxanthine, xanthine, urate, and uracil may suggest enhanced nucleotide degradation in the affected muscle. Elevated levels of these metabolites can also contribute to altered redox homeostasis as the generation of xanthine and urate are accompanied by the production of hydrogen peroxide (H2O2). Furthermore, previous studies demonstrated that purine catabolism may be a component of the homeostatic response of mitochondria to oxidative stress [52].

## Conclusions

The findings in this study suggest that Wooden Breast disease in commercial chickens is accompanied by a unique biochemical signature that may have diagnostic potential. Future studies that examine the plasma or serum from affected and unaffected animals may confirm the utility of these metabolites as non-invasive biomarkers. A predictive accuracy of 100% by random forest analysis (RFA) for detecting affected cases is remarkable considering that the chickens used in this study were sampled from two genetically distinct purebred lines and one commercial broiler population (more information about genetic diversity in these populations can be found in Fu et al., 2015 [53]).

Although the exact etiology of this novel muscle disease is currently unknown, our study provides important information regarding metabolic pathways and biochemicals involved in the pathogenesis of Wooden Breast disease. The presence of oxidative stress in affected muscle, which has been previously hypothesized through gene expression [6] and microscopic analyses [1], is well supported by our metabolomics findings. In addition, our findings provide mechanistic insights into metabolic pathways potentially contributing to oxidative stress in affected muscle. The results confirm altered glucose metabolism in affected chickens, which has been previously postulated based on findings from a RNA-sequencing experiment [6]. Finally, our results suggest that vitamin C supplementation may help lower the incidence of the Wooden Breast myopathy in modern broiler chickens. Future research using different levels of vitamin C in the broilers’ diet may help elucidate the usefulness of vitamin C supplementation.

## Supporting Information

S1 File The spectral entries for the metabolites identified in our sample set. (XLSX)

S2 File The complete dataset received from Metabolon Inc. comprised a total of 282 compounds of known identity. All original data and log-transformed median scaled values are provided.
(XLSX)

S3 File Glycogen measurements in Wooden Breast-affected and unaffected samples. (XLSX)
